# Reliability of the Italian Version of the Fugl-Meyer Upper Extremity Scale Administered Remotely

**DOI:** 10.3390/jcm13247750

**Published:** 2024-12-19

**Authors:** Francesca Falchini, Marco Germanotta, Alessio Fasano, Laura Cortellini, Sabina Insalaco, Valeria Cipollini, Dionysia Papadopoulou, Irene Giovanna Aprile

**Affiliations:** IRCCS Fondazione Don Carlo Gnocchi ONLUS, 50143 Florence, Italy; mgermanotta@dongnocchi.it (M.G.); lcortellini@dongnocchi.it (L.C.); sinsalaco@dongnocchi.it (S.I.); vcipollini@dongnocchi.it (V.C.); dpapadopoulou@dongnocchi.it (D.P.); iaprile@dongnocchi.it (I.G.A.)

**Keywords:** stroke, rehabilitation, telerehabilitation, telehealth, validation, Fugl-Meyer Assessment, upper extremity

## Abstract

**Background/Objectives:** Despite the increase in home-based rehabilitation, outcome measures for telerehabilitation are still underdeveloped. The Fugl-Meyer Assessment (FMA) is one of the most widely used tools for evaluating post-stroke motor deficits, with the upper extremity component (FMA-UE) recommended for assessing motor deficits of the arm. This study aims to examine the intrarater and interrater reliability of the Italian version of the FMA-UE, administered remotely via video conferencing during a robotic telerehabilitation program. **Methods:** Twenty stroke patients participated and underwent 20 sessions of remote upper limb rehabilitation with a robotic device. In-person evaluations were conducted before (T0) and after (T1) treatment, with additional remote assessments throughout. The study evaluated both intrarater and interrater reliability using Intraclass Correlation Coefficients (ICC) and Bland–Altman plots, classifying reliability as excellent for scores above 0.90. **Results:** Bland–Altman analysis showed no systematic variance for both intrarater and interrater reliability of the FMA-UE scale. Excellent reliability was found with intrarater ICC = 0.972 and interrater ICC = 0.981. Sections A and C of the FMA-UE showed excellent intrarater reliability, while sections B and D had satisfactory results. Both intrarater and interrater reliability analysis of the total score of the FMA-UE scale also showed strong agreement with Cohen’s Kappa values above 0.70. **Conclusions:** The findings suggest that the remote administration of the FMA-UE scale is a reliable tool for assessing upper limb motor function in stroke patients, supporting its use in telerehabilitation settings.

## 1. Introduction

Stroke is acknowledged as the second largest cause of mortality in adults, as well as the major cause of physical disability [1]. The number of stroke survivors has grown in recent years, with a consequent increase in the demand for rehabilitation services [2]. Only 12% of stroke patients have a full recovery of the functioning of the upper limbs six months after the stroke event [3]. As defined by the International Classification of Functioning, Disability, and Health (ICF) [4], upper limb motor deficits persist in the remaining 88%, having a negative influence on their level of activities [5,6,7] and involvement [8].

Robotic and technological rehabilitation has emerged as an effective solution to deliver high therapy doses to patients [9]. Technology-based rehabilitation has proven to be an excellent alternative to conventional ones in treating stroke patients, particularly during the COVID-19 pandemic with the diffusion of telerobotic rehabilitation, already promoted and implemented in physical medicine and rehabilitation prior to the COVID-19 outbreak. Telerehabilitation refers to therapeutic interventions that are delivered outside of a hospital setting, usually in the domestic or community settings, bringing advanced rehabilitation technology to the comfort of patients’ homes. This approach allows individuals to participate in personalized therapeutic activity programs [10,11]. Therefore, these solutions could be employed not solely for exceptional situations such as pandemics, but also to provide care to people who experience limitations in reaching appropriate healthcare resources due to geographical or other constraints [12].

In this context, it is crucial to develop novel methods for determining telerehabilitation protocols’ efficacy on patients with non-in-person assessments. While there is a growing body of literature on home rehabilitation, including the emerging role of technology, as evidenced by the 2022 review by Toh et al. [13], outcome measures for the use of telerehabilitation are currently underdeveloped and understudied.

The Fugl-Meyer Assessment (FMA) is the most commonly used tool for evaluating stroke patients, with the upper extremity motor component (FMA-UE) being a highly recommended and specific method outlined in the guidelines of the American Heart Association/American Stroke Association for assessing motor deficits in the arm [14]. It has been demonstrated that it is a reliable tool both for in-person [15] and through video-recorded assessment [16].

The capacity to conduct dependable assessments remotely enables patients who experience challenges in accessing healthcare facilities due to age or mobility to be monitored more efficiently by their healthcare providers. Furthermore, this approach could potentially result in cost savings for both the patient and the healthcare facility.

Several studies have addressed this issue. Some have attempted to deliver the FMA scale via teleconsultation by applying some modifications, in particular, they selected precise subscales to deliver or modified the instruction for certain sections [17,18,19]. While other assessments of the FMA scale have been administered via virtual reality [20] or sensors [21], these are too expensive and impractical for assessments performed in the patient’s home with only the help of a caregiver. To date, no attempt has been made to study the reliability of the remotely administered Italian version of the FMA scale, in particular after a remote robotic rehabilitation.

In our prior experience with a multicenter randomized trial [22], the Upper Extremity Fugl-Meyer Assessment was recorded on video to ensure consistent and reliable evaluations of patients at different time points. This method enabled trained therapists to conduct blinded assessments, thereby enhancing the objectivity and standardization of the evaluations. Furthermore, this approach demonstrated that the FMA-UE could be effectively assessed via video without any challenges in interpretation, highlighting its feasibility for remote assessment.

Therefore, the aim of this investigation was to examine both intrarater and interrater reliability of the Italian version of the FMA-UE scale administered remotely via video conferencing systems after a robotic telerehabilitation protocol.

## 2. Materials and Methods

### 2.1. Study Design and Participants

This methodological study is part of a larger interventional clinical study performed between May and August 2021. The cohort study was conducted in accordance with the Declaration of Helsinki, the principles of Good Clinical Practice, local legislation in participating countries and was approved by the Ethics Committee “Comitato Etico Lazio 1” on 6 May 2021 (610/CE Lazio 1). The research has been recorded on ClinicalTrials.gov under the identifier code (NCT05250934).

The original clinical study aimed to assess the feasibility of a rehabilitation treatment in a home setting based on a teleconsultation, telemonitoring, and robotic telerehabilitation system. This system employed the Icone (Haexel) robot and integrated sensor technology for neurological patients, with the objective of overcoming the limitations imposed by the ongoing pandemic.

**Figure 1:**
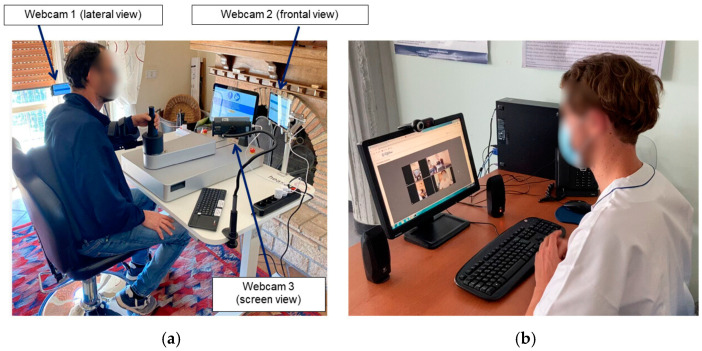
Rehabilitation setting: (**a**) depicts the home setting with the upper limb rehabilitation robot; (**b**) shows how the therapist followed the remote rehabilitation sessions.

The patients underwent a telerobotic rehabilitation treatment which consisted of 1 h daily robotic upper limb treatments for a total of 20 sessions. During the treatment, two specifically positioned webcams (one for the patient’s sagittal plane and one for the frontal plane) allowed for the observation of the patient while performing movements, ensuring that correct postures were maintained and that potentially unlikely hazardous situations did not occur. A third webcam was also used to display the screen, enabling monitoring of the patient’s activities and the feedback displayed on the screen (Figure 1).

The study participants were recruited from the Fondazione Don Carlo Gnocchi Center Santa Maria della Provvidenza patient cohort in Rome from May 2021 to August 2021. Twenty patients with stroke outcomes and a latency from the acute event greater than three months were recruited for the study, according to the following inclusion criteria: (a) Stroke outcomes (both hemorrhagic and ischemic) documented by Magnetic Resonance Imaging (MRI) or Computed Tomography (CT) imaging; (b) age between 18 and 85 years; (c) latency from the event greater than 3 months; (d) upper limb hemiparesis (FMA-UE ≤ 58); (e) trunk control ability; (f) patients previously admitted to the Santa Maria della Provvidenza center for upper limb rehabilitation treatment (residential, semi-residential, or outpatient); (g) availability of a caregiver who can support and supervise the patient during telerehabilitation sessions. While patients exhibiting (a) upper extremity stiffness (Modified Ashworth Scale score of 4); (b) cognitive deficits that could impede comprehension of rehabilitation instructions (MMSE score of less than 22); (c) behavioral disorders that could impede therapeutic activity; (d) other orthopedic or neurological complications that could impede the rehabilitation protocol, and (e) inability or unwillingness to provide informed consent, were excluded.

### 2.2. Evaluation Process

**Figure 2:**
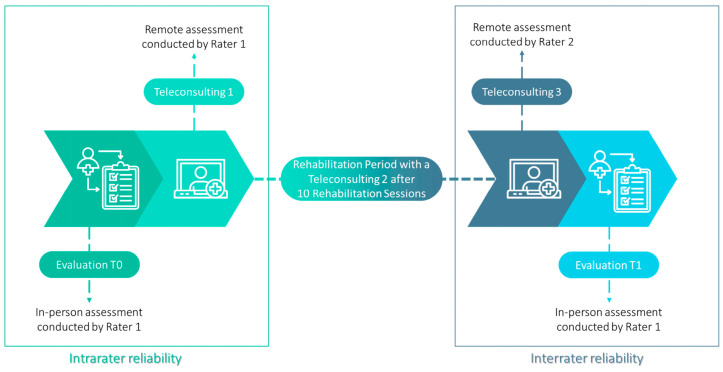
Evaluation steps of the patients participating in the study.

Each participant underwent two in-person evaluation sessions (at the clinical center), after the enrollment phase (T0) and at the end of the rehabilitation sessions (T1), and three evaluations through teleconsultation, administered via video conferencing. The first teleconsultation occurred right at the beginning of the rehabilitation therapy (Tele1), while the other two evaluations (Tele2, Tele3) were performed after ten and twenty sessions of robotic telerehabilitation, as part of the scheduled teleconsultations. The in-person evaluations were performed by the same rater (R1). The remote evaluations were performed as follows: the initial remote evaluation (Tele1) and one of the evaluations conducted after the initial ten rehabilitation sessions (Tele2) were carried out by the same evaluator as the evaluation at T0 (i.e., R1). In contrast, the final remote evaluation (Tele3) was conducted by a different evaluator (R2) following the 20th rehabilitation session. Raters were blinded to the patients’ treatment, i.e., physical therapists treating patients were not involved in the assessments. The evaluation steps of the patients participating in the study are illustrated in Figure 2. During teleconsultation, participants were instructed to position themselves in a pre-arranged environment for the assessment. It is important to note that the remote assessment of the FMA-UE was conducted using the same cameras employed during the rehabilitation sessions. Additionally, the patient was required to be seated near the robot desk to ensure consistency. The assessment environment was kept identical for evaluations at time point T0 and T1 and also between Tele1 and Tele3 while it differs between the clinical and home settings, particularly for the assessments conducted at T0 and Tele1, as well as at Tele3 and T1. All assessments were performed with participant consent.

### 2.3. Clinical Evaluation Scale

Patients were clinically evaluated using the Italian version of the FMA-UE (i.e., A–D sections). In administering the FMA-UE, both in-person and remote assessments, items describing reflex activities (osteo-tendon reflexes) were excluded due to the inability to obtain clinically relevant data remotely. Therefore, the maximum total score that could be obtained was equal to 60 points, rather than 66. Sections H (sensitivity) and J (movement and joint pain during passive movement) were excluded from the analysis, as their remote administration was deemed unreliable. Furthermore, the physiotherapist required the assistance of the caregiver in testing the patient’s wrist movements, hand movements, and tactile and proprioceptive sensitivity during the assessment sessions.

### 2.4. Statistical Analysis

The absolute test–retest reliability was evaluated by comparing the index data obtained during the test sessions via paired sample Student’s *t*-tests and Bland–Altman plots. Data normality for the total score of the FMA-UE (T0 vs. Tele1 and Tele3 vs. T1) was assessed through Shapiro–Wilk test.

The intrarater and interrater reliability of the FMA-UE scores was evaluated using the Intraclass Correlation Coefficient (ICC) with a 95% confidence interval (CI). For the intrarater reliability, a two-way mixed-effects, absolute agreement, single rater/measurement model was selected, while for the interrater reliability, a two-way random-effects, absolute agreement, single rater/measurement model was employed. The reliability of the data were classified as excellent (ICC > 0.90), good (0.75 < ICC ≤ 0.90), moderate (0.5 < ICC ≤ 0.75), or poor otherwise [23]. In order to assess intrarater reliability, data from patients acquired at time points T0 and Tele1 were compared. Similarly, to evaluate interrater reliability, data obtained at time points Tele3 and T1 were analyzed. Additionally, we analyzed the reliability of both in-person and remote FMA-UE assessments through the weighted Cohen’s Kappa ($\kappa_{w}$).

The statistical analysis was performed using the SPSS software (version 28, IBM Corp., Armonk, NY, USA).

## 3. Results

Twenty patients with chronic phase stroke outcomes were enrolled in the study and were assessed at baseline. Two patients did not complete the planned treatment due to causes unrelated to the study; therefore, 18 patients completed the 20 treatment sessions and were evaluated at the end of treatment. In addition, the end-of-treatment assessment for one patient was deemed unreliable due to the patient’s uncooperative behavior, therefore it was excluded from the interrater reliability analysis.

The demographical characteristics of the 20 enrolled patients are reported in Table 1.

The clinical characteristics are detailed in Table 2, which highlights an average latency of 39 months from the acute event.

Shapiro–Wilk test confirmed the normality of FMA-UE total score data distribution.

Paired sample *t*-test results showed no significant differences between the FMA-UE total score obtained at T0 and the FMA-UE total score obtained at Tele1 (*p* = 0.492) and between the FMA-UE total score obtained at Tele3 and the FMA-UE total score obtained at T1 (*p* = 0.130).

**Figure 3:**
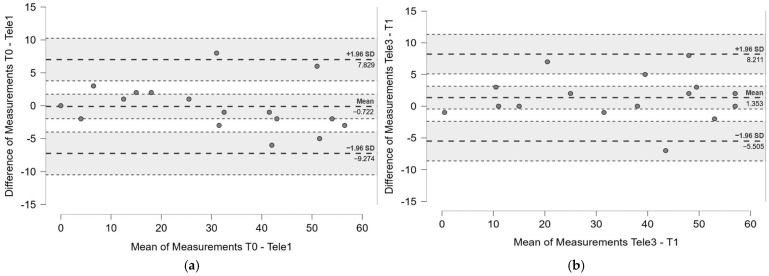
Bland–Altman plot of the Fugl-Meyer Assessment Upper Extremity (FMA-UE) scores for (**a**) intrarater analysis for the assessments at time point T0 and Tele1; (**b**) interrater analysis for the assessments at time point Tele3 and T1. The gray bands indicate the 95% confidence intervals. The central dotted line represents the mean difference, while lower and upper dotted lines indicate, respectively, mean difference − 1.96 SD and the mean difference + 1.96 SD.

No systematic variance was found through Bland–Altmann analysis in both the intrarater reliability (Figure 3a) and interrater reliability (Figure 3b) analyses of the FMA-UE. The mean differences and limits of agreement obtained with Bland–Altman analysis are displayed in Table 3.

As shown in Table 4, both intrarater and interrater analysis yielded excellent reliability of the deployment of the FMA-UE scale (ICC_intrarater_ = 0.972, 95% CI = 0.927 to 0.989; ICC_interrater_ = 0.981; 95% CI = 0.952 to 0.993).

Upon closer examination of the individual sections of the upper extremity FMA scale, it can be observed that for the intrarater reliability analysis sections A and C yielded equally excellent results, while sections B and D demonstrated satisfactory outcomes. On the other hand, we find excellent ICC values for interrater reliability in all sections and for the overall score.

Intrarater and interrater reliability evaluated using the weighted Cohen’s Kappa ($\kappa_{w}$), yielded also excellent results. In both cases, the $\kappa_{w}$ values exceeded 0.70 for the total FMA-UE (A-D sections), indicating a high level of reliability (Table 5).

## 4. Discussion

The present study aims to test the intrarater and interrater reliability of the remote FMA-UE in stroke patients.

The reliability of upper limb motor function measurements was evaluated through the use of Bland–Altman analysis and intraclass correlation indices (ICC). No significant systematic variation was identified in the intrarater and interrater reliability of the FMA-UE scale, with non-significant mean differences. ICC values indicated excellent reliability for both intrarater and interrater assessments.

Specifically, sections A and C demonstrated excellent results for intrarater reliability, while sections B and D exhibited satisfactory results. With regard to interrater reliability, all sections demonstrated excellent ICC values. Furthermore, reliability according to weighted Cohen’s Kappa confirmed a high level of reliability in both intrarater and interrater ratings.

Therefore, the FMA-UE scale was found to be highly reliable for assessing upper limb motor function in post-stroke patients, both when administered by the same assessor and by different assessors remotely.

As telerehabilitation has gained momentum in recent years, it has become increasingly important to examine the reliability of remote assessment scales. Several studies have already assessed different scales remotely [16,17,19,20,24], and it has been seen that the results in many cases are good if not excellent.

In particular, some of them have sought to assess the reliability of the remotely administered FMA-UE scale, typically by introducing modifications to its administration [17,19]. Specifically, this has entailed either eliminating the reflex section [19], a decision based on the studies of Woodbury et al. [25,26], in which the authors found that reflex testing did not contribute to the FMA-UE scores, or eliminating both reflex and coordination tasks [17].

A comparison of our results with those of studies that have analyzed the reliability of the FMA-UE remotely reveals that, in the majority of cases, the results demonstrate excellent levels of reliability, as observed in our findings.

This accomplishment is significant because it furnishes telerehabilitation and remote rehabilitation, in general, with a dependable instrument for non-presence assessment of patients, thus obviating the necessity for them to attend the clinical facility for each assessment, including those of an intermediate nature. This reinforces the notion that remote assessments enhance accessibility by reducing the barriers commonly encountered in traditional trial design [27]. In light of the insights gained from this investigation, the remote FMA scale will be considered for future employment as an assessment instrument within the context of a multicenter pragmatic trial investigating telerehabilitation models based on enhanced home environment, incorporating digital technologies, in a large cohort of patients who have experienced a stroke (as, for example, the STROKEFIT4@HOME pragmatic trial).

In our study, no issues were encountered with items that required the input of the caregiver, nor were there any connectivity issues that could have affected the assessment itself. This is due in part to the fact that the robot already provided a reliable communication system of its own, comprising two devices that could show the patient’s movements and a stable connection that would allow for calls to be made regardless of the location of the patient’s home.

Consequently, despite the time required for proper setup, the administration time was comparable to the time required to administer the scale remotely.

It is evident that this may prove to be a limitation in other scenarios where clinicians and therapists can rely solely on the availability and accessibility of the patient’s home network, as seen in other studies [18,19].

The limitation of this study is the relatively small sample size, which is a consequence of the fact that it is a secondary analysis of a study that focused on telerehabilitation. Further investigation with a more expansive subject group will enable more definitive findings to be produced; nevertheless, we believe that the quality of these findings will be on par with those presented in this paper.

## 5. Conclusions

This study demonstrated that the Italian version of the Fugl-Meyer Assessment of Upper Extremity (FMA-UE) scale can be reliably utilized to assess upper extremity motor function in stroke patients, even when administered remotely via video conferencing as part of a robotic telerehabilitation protocol. The results indicate excellent consistency of ratings, both across different assessors and for the same assessor over time.

The presented evidence lends support to the utilization of the FMA-UE in telerehabilitation settings, where the necessity for remote assessment is needed. The capacity to conduct precise and dependable assessments without the patient’s physical presence represents a substantial advancement in post-stroke rehabilitation access, particularly in contexts where healthcare resources are scarce or patient mobility is constrained. Further research could investigate the applicability of this scale in other rehabilitation settings and in larger and more diverse patient populations.

## Figures and Tables

**Table 1:** Demographical characteristics of enrolled patients.

Age	66 ± 9 (52–82)
Sex	11 men, 9 women
Dominant side	18 right-handed, 2 left-handed
Height (cm)	165.5 ± 10.3 (150–182)
Weight (kg)	68.1 ± 13.8 (42–90)
BMI	24.8 ± 4.4 (17.9–33.3)
Schooling (years)	11.5 ± 5.5 (5–18)

**Table 2:** Clinical characteristics of enrolled patients.

Latency since acute event (months)	39.0 ± 12.7 (3–39)
Stroke type	14 ischemic, 6 hemorrhagic
Hemiparesis	10 hemidestrians, 10 hemisynisters
Aphasia	1
Neglect	3
FMA-UE motor function	32.9 ± 17.4 (4–59)
FMA-UE sensitivity	9.3 ± 3.0 (1–12)
Pain (NRS)	2.6 − 3.1 (0–8)

**Table 3:** Bland–Altman mean differences and limits of agreement.

	Bland–Altman Intrarater			Bland–Altman Interrater		
	Value	95% Confidence Interval		Value	95% Confidence Interval	
		Lower Bound	Upper Bound		Lower Bound	Upper Bound
Mean difference + 1.96 SD	7.829	4.071	11.587	8.211	5.095	11.327
Mean difference	−0.722	−2.892	1.447	1.353	−0.446	3.152
Mean difference − 1.96 SD	−9.274	−13.032	−5.516	−5.505	−8.621	−2.389

**Table 4:** Intraclass correlation coefficients both for intrarater and interrater reliability of the whole Fugl-Meyer upper extremity assessment and of the single sections.

	Intrarater Reliability			Interrater Reliability		
	ICC (3,1)	95% Confidence Interval		ICC (2,1)	95% Confidence Interval	
		Lower Bound	Upper Bound		Lower Bound	Upper Bound
FMA-UE Section A	0.951	0.875	0.982	0.986	0.960	0.995
FMA-UE Section B	0.822	0.585	0.929	0.938	0.849	0.975
FMA-UE Section C	0.938	0.843	0.976	0.927	0.824	0.971
FMA-UE Section D	0.820	0.581	0.929	0.917	0.802	0.967
FMA-UE (Sections A–D)	0.972	0.927	0.989	0.981	0.952	0.993

**Table 5:** Intraclass correlation coefficients both for intrarater and interrater reliability of the entire Fugl-Meyer upper extremity assessment and of the single sections.

	Intrarater Reliability					Interrater Reliability				
	$\kappa_{w}$	Lower Bound	Upper Bound	SE	*p*	$\kappa_{w}$	Lower Bound	Upper Bound	SE	*p*
FMA-UE Section A	0.804	0.699	0.908	0.053	<0.001	0.830	0.751	0.908	0.040	<0.001
FMA-UE Section B	0.627	0.439	0.816	0.096	<0.001	0.782	0.666	0.898	0.059	<0.001
FMA-UE Section C	0.735	0.591	0.879	0.074	<0.001	0.723	0.547	0.899	0.090	<0.001
FMA-UE Section D	0.640	0.443	0.836	0.100	<0.001	0.806	0.638	0.974	0.086	<0.001
FMA-UE (Sections A–D)	0.815	0.734	0.896	0.045	<0.001	0.835	0.750	0.919	0.043	<0.001

## Data Availability

The data that support the findings are available from the first and corresponding author on reasonable request.
